# Undiagnosed type 2 diabetes is common – intensified screening of established risk groups is imperative in Sweden: the SDPP cohort

**DOI:** 10.1186/s12916-024-03393-0

**Published:** 2024-04-19

**Authors:** Hrafnhildur Gudjonsdottir, Per Tynelius, Nouha Saleh Stattin, Diego Yacamán Méndez, Anton Lager, Boel Brynedal

**Affiliations:** 1 Centre for Epidemiology and Community Medicine, Region Stockholm, Stockholm, Sweden; 2 Department of Global Public Health, Karolinska Institutet, Stockholm, Sweden; 3 Academic Primary Healthcare Centre, Stockholm, Sweden; 4 Department of Neurobiology, Care Sciences and Society, Division of Family Medicine and Primary Care, Karolinska Institutet, Huddinge, Sweden

**Keywords:** Type 2 diabetes, Undiagnosed, Healthcare, Risk factors, Screening, Public health

## Abstract

**Background:**

Undiagnosed type 2 diabetes (T2D) is a global problem. Current strategies for diagnosis in Sweden include screening individuals within primary healthcare who are of high risk, such as those with hypertension, obesity, prediabetes, family history of diabetes, or those who smoke daily. In this study, we aimed to estimate the proportion of individuals with undiagnosed T2D in Stockholm County and factors associated with T2D being diagnosed by healthcare. This information could improve strategies for detection.

**Methods:**

We used data from the Stockholm Diabetes Prevention Programme (SDPP) cohort together with information from national and regional registers. Individuals without T2D aged 35–56 years at baseline were followed up after two ten-year periods. The proportion of diagnosed T2D was based on register information for 7664 individuals during period 1 and for 5148 during period 2. Undiagnosed T2D was assessed by oral glucose tolerance tests at the end of each period. With logistic regression, we analysed factors associated with being diagnosed among individuals with T2D.

**Results:**

At the end of the first period, the proportion of individuals with T2D who had been diagnosed with T2D or not was similar (54.0% undiagnosed). At the end of the second period, the proportion of individuals with T2D was generally higher, but they were less likely to be undiagnosed (43.5%). The likelihood of being diagnosed was in adjusted analyses associated with overweight (OR=1.85; 95% CI 1.22–2.80), obesity (OR=2.73; 95% CI 1.76–4.23), higher fasting blood glucose (OR=2.11; 95% CI 1.67–2.66), and self-estimated poor general health (OR=2.42; 95% CI 1.07–5.45). Socioeconomic factors were not associated with being diagnosed among individuals with T2D. Most individuals (>71%) who developed T2D belonged to risk groups defined by having at least two of the prominent risk factors obesity, hypertension, daily smoking, prediabetes, or family history of T2D, including individuals with T2D who had not been diagnosed by healthcare.

**Conclusions:**

Nearly half of individuals who develop T2D during 10 years in Stockholm County are undiagnosed, emphasizing a need for intensified screening of T2D within primary healthcare. Screening can be targeted to individuals who have at least two prominent risk factors.

**Supplementary Information:**

The online version contains supplementary material available at 10.1186/s12916-024-03393-0.

## Background

Type 2 diabetes (T2D) is a common and chronic metabolic disease responsible for a considerable disease burden. In line with global estimates, the prevalence in Sweden is increasing, representing a public health challenge [1]. Individuals with T2D have a twofold increase in mortality [2], and a higher incidence of cardiovascular diseases [3, 4]. Treatment can effectively prevent morbidity and mortality if initiated at an early stage [5–7]. Many individuals already present some degree of irreversible micro- and macrovascular complications at the time of diagnosis [8, 9].

Identifying T2D at an early stage is challenging. People with T2D are often asymptomatic initially, which indicates that screening is needed to detect incident cases. However, population-wide screening is not effective in relation to mortality and is therefore not recommended [10]. Instead, it is recommended that individuals at risk, such as those with hypertension, obesity, prediabetes, family history of diabetes, or those who smoke daily, are screened for T2D when in contact with primary healthcare [11]. However, the evidence supporting which risk groups should be screened is limited. Alarmingly, global meta-analyses have shown that nearly 50% of individuals with T2D were undiagnosed in 2021 [12]. Two older cross-sectional Swedish studies found that nearly half of those with T2D were undiagnosed [13, 14]. But it is unknown if people diagnosed with T2D had it for a longer time, leading to more complications which enabled detection by healthcare. Following up a cohort of T2D free individuals in Sweden would enable accurate and updated estimates of the proportion of undiagnosed T2D.

It is unknown whether individual or social structural factors relate to the likelihood of being diagnosed when having T2D in Sweden. Acquiring knowledge on factors associated with undiagnosed T2D in our healthcare system could facilitate the implementation of processes to improve early detection. Previous studies indicate that individuals with low risk of having T2D are not screened, such as the reported association between being undiagnosed, and younger age, and not having a family history of T2D [15, 16]. Other findings suggest that screening is preferentially *not* performed in risk groups, such as the association between not being diagnosed and higher BMI, low physical activity, high metabolic risk, and male sex [15–17]. Not regularly seeing a doctor and living alone has also been shown to be associated with undiagnosed T2D [16]. Previous European studies did not find an association between socioeconomic factors and diagnosis status [15, 16, 18]. However, social inequalities in the quality of care of other conditions has been shown in Swedish populations [19–21].

We hypothesize that undiagnosed T2D is common in Sweden and that diagnosis status might be associated with individual and social structural factors. We aim to estimate the proportion of individuals who develop T2D over ten years but remain undiagnosed in Stockholm County and the factors associated with diagnosis status.

## Methods

### Data sources

In this study, we used data from the Stockholm Diabetes Prevention Programme (SDPP) cohort, a population-based, longitudinal study with repeated screening for T2D integrated with information from national and regional registers. This allows for reliable estimates of the proportion of individuals who develop T2D but are not diagnosed, as well as factors associated with being undiagnosed.

#### The Stockholm Diabetes Prevention Programme (SDPP) cohort

The SDPP cohort is described in detail elsewhere [22]. In this study, we included individuals who did not have a T2D diagnosis at baseline in 1992–1998 and participated in the clinical examinations. Everyone was born in Sweden, and close to half had a family history of diabetes. They were invited for a clinical examination at baseline (7949 participants), after ten years (5711 participants), and after twenty years (3627 participants). During the clinical examinations, their health, life situation, and diabetes status were examined through physical examinations and extensive questionnaires, and a fasting blood sample was drawn. Blood pressure, systolic and diastolic (mmHg), was measured with an aneroid sphygmomanometer while participants were in a sitting position. Oral glucose tolerance tests (OGTTs) were performed on all participants at baseline and on all who did not self-report diabetes at the follow-ups, where 75 g of glucose was dissolved in 2.5-3.0 L water and consumed by the participant. An additional blood sample was drawn after 2 hours.

#### Register data

Participants' sociodemographic and health-related information, including diabetes status, was retrieved by register linkages using the unique personal identity number assigned to all Swedish citizens. We used information on diagnoses from the National Diabetes Register, the Stockholm Regional Healthcare Data Warehouse (VAL), and the National Patient Register. The National Diabetes Register was established in 1996 and has nationwide coverage. It holds data on people aged 18 and older with diabetes [23]. VAL is an administrative database held by Region Stockholm, which is responsible for all publicly financed healthcare in Stockholm County. Diagnoses and healthcare utilization from primary care have been available since 2013, from specialist outpatient care since 2001 and from inpatient hospital care since 1993 [24]. The National Patient Register administered by The National Board of Health and Welfare covers all national inpatient care from 1987 onwards, and since 2001, it also includes all specialized outpatient visits to a doctor [25]. In addition, we used information from the National Prescribed Drug Register that contains information on all prescribed drugs dispensed at pharmacies in Sweden since 2005 [26]. We retrieved sociodemographic information from Statistics Sweden’s registries. The Longitudinal Integration Database for Health Insurance and Labour Market Studies (LISA) holds data since 1990 and combines sociodemographic data from the labour market, educational and social sectors [27]. Information on the date of death and migration was retrieved from the Total Population Register [28].

### Design of periods and eligibility

**Fig. 1:**
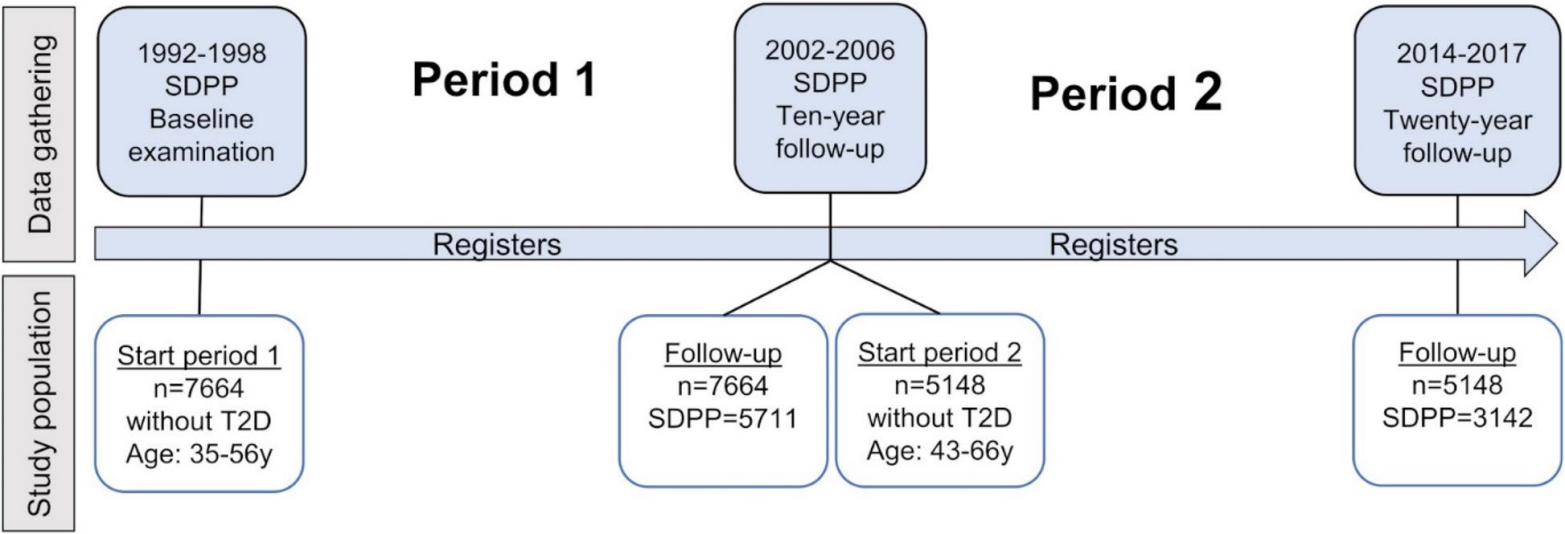
Source and time of data gathering and the number of eligible participants.SDPP: The number of eligible individuals who participated in the clinical examinations at follow-ups in the SDPP cohort

We followed-up the individuals after two periods with an approximate duration of ten years each (Fig 1). Period 1 started after the baseline examination and ended with the 10-year follow-up, and period 2 began at the ten-year follow-up and ended with the 20-year follow-up. The eligible individuals for each period were those who participated in the examination at the start of the period and were free of T2D at this point and whose T2D status could be assessed at the time of follow-up. The individuals who chose not to attend follow-ups could still be followed up within registries to assess diagnosis of T2D. Those who died or moved abroad were excluded, regardless of T2D diagnosis (N: 156, of whom none had known T2D, and 274, of whom 23 had T2D, in periods 1 and 2, respectively). This resulted in 7664 and 5148 included individuals in periods 1 and 2, respectively.

### Variables

#### Outcome

Those with T2D identified through registers during the period follow-up or self-reported T2D through questionnaires at follow-up were categorized as diagnosed T2D. The reference sample included everyone in the cohort who was alive and lived in Sweden at the time of the follow-up (N: 7664 and 5148 for periods 1 and 2, respectively, Fig. 1). T2D diagnoses were retrieved from registries (The National Diabetes Register, VAL, The National Patient Register) as code E11 according to the International Classification of Diseases 10^th^ revision (ICD-10). Additionally, a minimum of four dispensed prescriptions of anti-diabetic drugs for over at least two years according to the Anatomical Therapeutic Chemical System (ATC) code A10 was assessed from the National Prescribed Drug Register and registered as diagnosed T2D. Everyone who had a T2D diagnosis according to register data and participated in follow-ups also self-reported T2D. Only seven individuals self-reported T2D and did not have a registered T2D diagnosis in our data during period 1 and eight in period 2 (included as diagnosed T2D).

Undiagnosed T2D was determined from OGTT screening results among individuals who attended the clinical examination at follow-up within the study (N: 5711 and 3142 for periods 1 and 2, respectively, Fig. 1). During OGTT, a T2D diagnosis was ascertained as the value of fasting plasma glucose ≥ 7.0 mmol/L and/or 2-hour post load plasma glucose ≥ 11.1 mmol/L. Undiagnosed T2D was defined as those who fulfilled T2D diagnosis criteria according to OGTT but did not belong to the group with diagnosed T2D.

#### Exposure

We selected potential factors associated with the chance of being diagnosed among individuals with T2D based on previous literature [15–18] as well as factors that might indicate inequality in healthcare. Exposures were assessed at the start of the periods (Fig 1). Age and sex were collected from the Total Population Register. Education was assessed from Statistics Sweden data on 7 levels and categorized as at most primary school (≤9 y), upper secondary school (12 y), or university or higher (>12 y).

Body weight and height were measured at each SDPP clinical examination, and BMI was calculated as kg/m^2^. BMI was categorized into normal weight (BMI<25 kg/m^2^), overweight (BMI=25–29.9 kg/m^2^), and obesity (BMI ≥ 30 kg/m^2^) [29]. Blood pressure was categorized into hypertension (systolic blood pressure ≥ 140 and/or diastolic ≥ 90) [30] and not hypertension. Fasting plasma glucose was analysed from blood samples and used as a continuous variable.

Additional exposures were collected from the questionnaires at the SDPP clinical examinations. Questions on smoking and snuff use had the response alternatives never, former, or current daily use and were categorized for analysis purposes into daily smoking or snuff use or not. Alcohol consumption was calculated in g/week from self-reported standard units and frequencies and hazardous alcohol consumption was defined as ≥120 g/week [31]. Physical activity was measured with the question “How physically active have you been during your spare time during the last year?” with the response alternatives sedentary, light physical activity, moderate physical activity, and moderate to vigorous physical activity. The cohabitation status question “Are you married or cohabiting?” was categorized as yes or no, and the self-reported general health question “How is your general health?” was categorized as good (very good, quite good or neither good nor bad) or bad (quite bad or very bad). The long-term illness question “Do you have any long-term illness, health problems following an accident, disability, or other persistent health problems?” was used as yes or no. Occupation was assessed using the question “State your current or previous occupation or job”, answers were manually encoded according to standard for Swedish occupational classification from 1987, and thereafter categorized as high and intermediate nonmanual employees or not according to Statistics Sweden’s categories [32]. A family history of diabetes was collected from the screening questionnaire and was confirmed by participants at the clinical examinations. Family history was classified as self-reporting one first-degree relative with T2D or two second-degree relatives with T2D [22].

### Statistical methods

The baseline characteristics of the whole study population and for those diagnosed with T2D during the study periods are presented as the mean and standard deviation or median and quintiles for continuous variables and as proportions for binary and categorical variables.

We calculated the proportion of diagnosed and undiagnosed T2D separately at the end of each time-period. The proportions of individuals with diagnosed, and undiagnosed T2D were compared in each period using chi-square tests. For sensitivity analysis, we estimated the proportion of diagnosed T2D where we included those who had developed T2D before they died or moved abroad during the study period. Furthermore, in another analysis, we assumed that everyone who either died or moved abroad during periods 1 or 2 had diagnosed T2D and estimated the proportion of diagnosed T2D during either period. We also calculated the proportion of diagnosed T2D if none of the above developed diagnosed T2D during the study period. Finally, we assumed that all lost to follow-up developed undiagnosed T2D, and that none of them developed undiagnosed T2D.

We estimated the proportion of individuals who developed T2D during the study and belonged to a risk group where screening for T2D is recommended. We included prediabetes, hypertension, obesity, daily smoking, or family history of diabetes and calculated the proportion of individuals with at least two of these risk factors. Based on this two-factor risk group we calculated the sensitivity and specificity of detecting individuals who would develop T2D within 10 years.

We used logistic regression to estimate odds ratios (ORs) with 95% confidence intervals (CIs) for the probability of being diagnosed by healthcare among individuals who developed T2D. Logistic regression models were fitted individually for each exposure, first as univariable models, second adjusted for sex and age, and third adjusted additionally for lifestyle factors (daily smoking, daily snuff use, hazardous alcohol consumption and physical activity). Based on the assumption of missing at random, we used Multivariate Imputation by Chained Equations (MICE) to impute missing data. The imputation model included all variables used in the subsequent analyses. We used 100 burn-ins and generated 20 datasets, and convergence was assessed from trace plots. In another analysis, we performed logistic regression models that only included complete cases of each variable. Stata version 17.0 [33] was used for statistical analysis.

## Results

### Proportion of individuals with diagnosed and undiagnosed T2D

At the end of the first period, 157 participants were found to have undiagnosed T2D, and 180 had diagnosed T2D. This gave a proportion of 2.3% for diagnosed T2D and 2.7% for undiagnosed T2D (Table 1). At the end of the second period, we identified 156 individuals with undiagnosed T2D and 335 individuals with diagnosed T2D, reflecting 6.5% diagnosed, and 5.0% undiagnosed.

**Table 1:** The proportion of individuals with diagnosed T2D, undiagnosed T2D, and the ratio of undiagnosed T2D during period 1 and 2

	Diagnosed T2D %	Undiagnosed T2D %	Ratio undiagnosed^b^ %
Period 1 (35–56-year-old^a^)	2.3(180/7664)	2.7(157/5711)	54.0 (2.7/5)
Period 2 (43–66-year-old^a^)	6.5 (335/5148)	5.0(156/3142)	43.5 (5/11.5)

Diagnosed T2D, Diagnosed by healthcare, undiagnosed T2D, Detected within SDPP

^a^At beginning of the period

^b^Ratio undiagnosed = (diagnosed within SDPP) / (diagnosed within SDPP + diagnosed by healthcare)

At the end of the first period, when the participants were 43–66 years old, the proportion of undiagnosed T2D was similar to that of diagnosed T2D (p value: 0.11, Table 1). At the end of the second period, when participants were 10–12 years older, T2D was more prevalent, and the proportion of diagnosed T2D was higher than that of undiagnosed T2D (6.5% vs 5.0%, *p* value: 0.027) (Table 1). On average, the rate of undiagnosed T2D during ten years was 48.8%.

In total, 5% of individuals with T2D (regardless of whether they were diagnosed or undiagnosed) had missing values for at least one variable, and the proportion of missing values ranged from 0.1% for education to 1.9% for grams of alcohol per week among individuals with T2D (Table 2). The characteristics of the 515 individuals with diagnosed T2D and the 313 individuals with undiagnosed T2D are presented separately (see Additional file 1: Table S1).

**Table 2:** Baseline characteristics of all participants, and the subgroup who developed type 2 diabetes during the study (diagnosed and undiagnosed)

	The full population *n*=7664	Individuals with T2D *n*=828	Missing^a^ *n*(%)
Sex			-
Women, %	61.1	46.0	
Age (years), mean (Sd)	47.0(4.9)	48.1(4.5)	-
BMI (kg/m2), mean (Sd)	25.6(3.9)	28.4(4.4)	3 (0.36)
Family history of type 2 diabetes, %	57.7	78.1	-
Highest attained education			1 (0.12)
University (>12y), %	33.5	24.7	
Upper secondary school (12y), %	47.9	48.4	
Primary school (9y), %	18.6	27.0	
High-and intermediate nonmanual employees, %	43.9	39.0	2 (0.24)
Fasting glucose (mmol/L), mean (Sd)	4.7(0.54)	5.2(0.62)	-
Systolic blood pressure, mmHg, mean (Sd)	122.6(15.8)	129.1(15.9)	7 (0.85)
Daily smoking, %	26.0	31.8	-
Daily snuff, %	7.8	10.4	-
Alcohol, g/week, median (25%-75%)	46.6 (18.2–91.6)	49.1 (17.1–109.4)	16 (1.9)
Physical activity			-
Sedentary, %	10.7	15.3	
Moderate, %	54.7	56.6	
Regular, %	26.9	23.0	
Active, %	7.8	5.1	
Living alone, %	16.3	15.3	5 (0.6)
Poor general health, %	2.0	4.0	-
Long term illness, %	27.7	33.7	8 (0.97)

*BMI* Body mass index, *T2D* Type 2 diabetes

^a^Missing values among individuals with T2D

### Association analyses

Based on data from 828 individuals with T2D, we investigated exposures associated with being diagnosed. The likelihood of being diagnosed by healthcare was in unadjusted analyses associated with older age, overweight and obesity, hypertension, and higher fasting plasma glucose (Table 3., model A) at the start of the period. These associations persisted as statistically significant after adjusting for age and sex (model B) and after additionally adjusting for lifestyle factors (model C), except for hypertension. In addition, we found an association with self-estimated poor general health when adjusting for age and sex (Model B), and for lifestyle factors (Model C). Education and employment were not associated with being diagnosed among individuals with T2D, nor was long-term illness.

**Table 3.** Odds ratios for the 828 individuals that developed T2D, diagnosed T2D compared to undiagnosed T2D for different risk factors at the beginning of the period

	**Model A**^**a**^	**Model B**^**b**^	**Model C**^**c**^
	OR (95% CI)	OR (95% CI)	OR (95% CI)
Sex	0.92 (0.69-1.22)	-	-
Age	1.04 (1.02-1.07)	-	-
Daily smoking	0.90 (0.66-1.24)	0.97 (0.70-1.33)	-
Daily snuff	0.84 (0.54-1.30)	0.90 (0.57-1.42)	-
Hazardous alcohol consumption	0.75 (0.55-1.02)	0.69 (0.50-0.97)	-
Physical activity			-
Sedentary	ref=1	ref=1	-
Moderate	0.96 (0.65-1.43)	0.91 (0.61-1.36)	-
Regular	1.31 (0.81-2.10)	1.27 (0.78-2.05)	-
Active	1.44 (0.73-2.80)	1.32 (0.67-2.61)	-
Education			
Primary school (9y)	ref=1	ref=1	ref=1
Upper secondary school (12y)	0.74 (0.52-1.04)	0.76 (0.54-1.07)	0.73 (0.51-1.03)
University (>12y)	0.93 (0.62-1.38)	0.92 (0.62-1.38)	0.87 (0.57-1.31)
High-and intermediate nonmanual employees	1.06 (0.80-1.42)	0.99 (0.73-1.33)	0.95 (0.71-1.29)
BMI			
kgm^2^	ref=1	ref=1	ref=1
25-29.99 kg/m^2^	1.77(1.18-2.66)	1.78 (1.18-2.69)	1.85 (1.22-2.80)
≥30 kg/m^2^	2.53 (1.66-3.87)	2.61 (1.70-4.01)	2.73 (1.76-4.23)
Family history of type 2 diabetes	1.05 (0.75-1.47)	1.09 (0.77-1.54)	1.09 (0.77-1.55)
Hypertension	1.41 (1.06-1.88)	1.26 (0.94-1.70)	1.30 (0.97-1.76)
Fasting glucose	2.13 (1.70-2.68)	2.05 (1.63-2.58)	2.11 (1.67-2.66)
Living alone	0.91 (0.62-1.32)	0.85 (0.58-1.26)	0.88 (0.60-1.29)
Poor general health	2.19 (0.99-4.87)	2.34 (1.04-5.24)	2.42 (1.07-5.45)
Long term illness	1.26 (0.94-1.68)	1.20 (0.89-1.61)	1.20 (0.89-1.62)

Reference: Undiagnosed T2D

^a^Crude

^b^Adjusted for sex and age

^c^Adjusted for sex, age, daily smoking, daily snuff, alcohol risk consumption and physical activity

**Fig.2:**
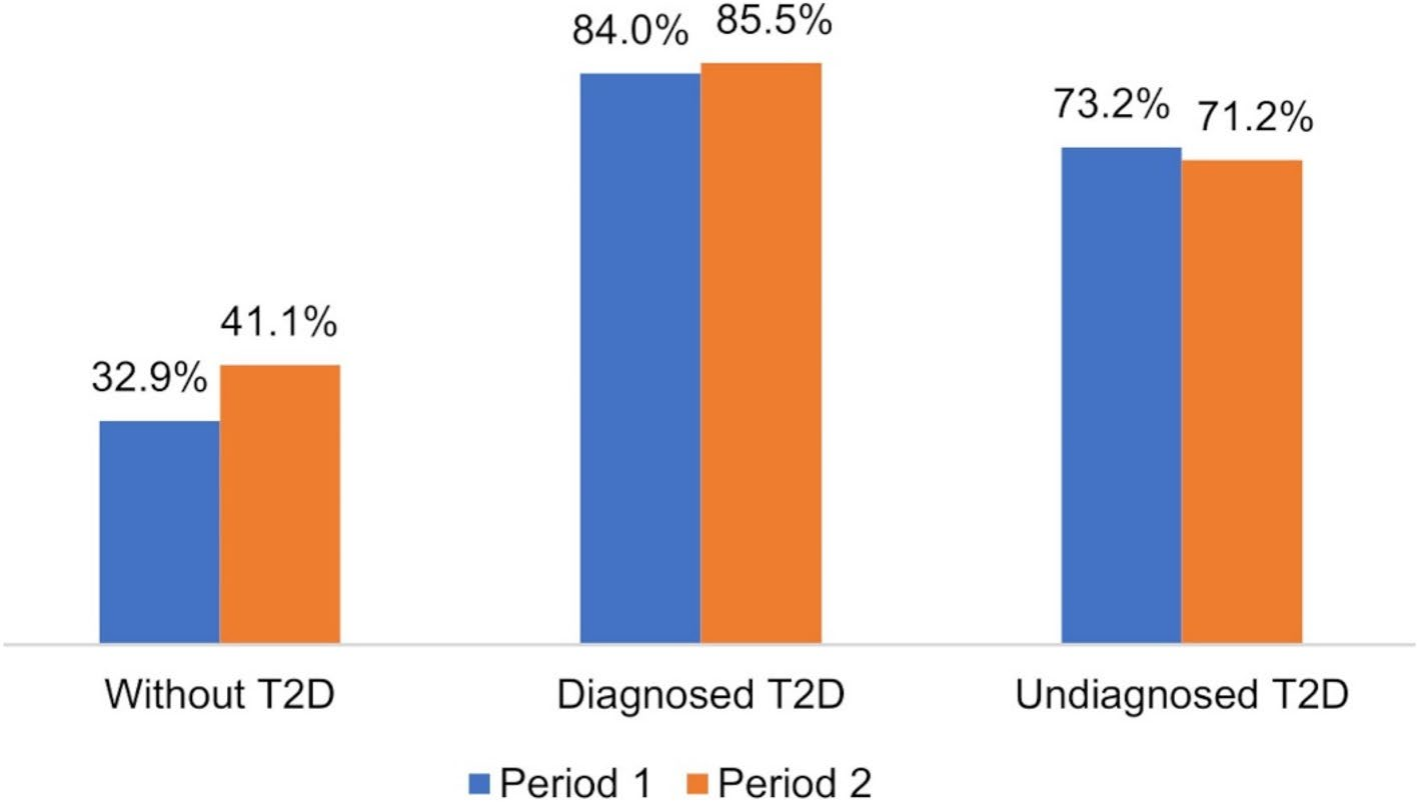
The proportion of individuals who belong to a risk group. The proportion of individuals with at least two risk factors (obesity, family history of diabetes, hypertension, daily smoking, or prediabetes) for T2D development among individuals without T2D, with diagnosed and undiagnosed T2D at the start of the period, in period 1 and period 2, respectively

Almost all of those who developed T2D belonged to an established risk group for T2D (97.8%); that is, they were obese, had a family history of diabetes, hypertension, prediabetes, or smoked daily. However, this is also common among those who did not develop T2D (77% and 81% in periods 1 and 2, respectively); that is, paying closer attention to all these factors would come at the price of very low specificity. A better specificity is achieved when defining the risk group as having two or more of the risk factors obesity, family history of diabetes, hypertension, prediabetes, or daily smoking: At least 71% of the individuals who developed T2D within 10 years belonged to this two-factor risk group, compared to a maximum of 41% among the individuals who would not develop T2D (Fig 2). This implies a sensitivity of 79% and 81% and a specificity of 67% and 59% in periods 1 and 2, respectively. In line with these results there was a significant association between belonging to a two-factor risk group and both diagnosed and undiagnosed T2D compared with normoglycemia in both periods (see Additional file 1: Table S2).

### Sensitivity analyses

When we included the 23 individuals who had developed T2D and died before follow-up in the second period, the proportion of diagnosed T2D increased from 6.5% to 6.9%. If we assume that everyone who we were not able to follow-up in registries (moved abroad or deceased, N: 156 and 274 in period 1 and 2 respectively) would have developed T2D and been diagnosed by healthcare, the proportion of diagnosed T2D increases markedly in both periods (see Additional file 1: Table S3). If we instead assume that no one who moved away or died developed diagnosed T2D, the proportion of diagnosed T2D becomes slightly lower than the observed proportion (see Additional file 1: Table S3). If we instead assume that everyone who was lost to follow-up would have developed undiagnosed T2D, the proportion of undiagnosed T2D increases strongly. Lastly, if we assume that everyone who was lost to follow-up did not develop undiagnosed T2D the proportion of undiagnosed T2D decreases (see Additional file 1: Table S3).

We also performed logistic regression where we only included the 789 complete T2D cases to compare with the multiple imputation analysis, but the results remained unchanged (see Additional file 1: Table S4).

## Discussion

The aim of this study was to provide estimates of the proportion of individuals who develop T2D over ten years but remain undiagnosed in Stockholm County and factors associated with being diagnosed among individuals with T2D. We used data from a cohort of men and women without T2D with repeated OGTT screening and register data. Our main results are that roughly half of those who develop T2D during 10 years are not diagnosed. The rate of diagnosis among individuals increased between the studies two periods, which might reflect both that participants grew older (as indicated by the association analysis), due to attrition, increased health awareness among study participants, and that the register coverage and healthcare screening might have increased slightly over time. Our results suggest that there is a need for intensified screening of T2D in Sweden. Over 90% of the population in Stockholm County visits primary care at least once during five years, and most of them visit primary care even several times per year [34]. This implies that primary healthcare has the potential to detect those at risk to prevent or delay disease progression with early intervention. To increase the ability for primary healthcare centres to screen for T2D they need additional resources, and current Swedish recommendations on a maximum number of patients cared for by each general practitioner might facilitate appropriate T2D screening. Raised awareness through risk communication to the public for public health purposes could also be part of a strategy for increased detection.

We found that undiagnosed T2D is more common (49%) in Stockholm County than in recent studies from other Nordic countries [17, 35, 36]. One reason might be the different methods used to estimate T2D. The results from other Nordic studies were based on single HbA1c measurements, which have limited sensitivity [37], implying that many patients with T2D remained undetected in the studies. Indeed, when we compared our HbA1c and OGTT results from the SDPP 20-year follow-up, we detected a sensitivity of 25% for one measurement of HbA1c ≥48 mmol/mol. Our results are in accordance with previous cross-sectional Swedish studies [13, 14] based on OGTT.

Being undiagnosed was associated with fewer known T2D risk factors, such as younger age and fewer metabolic risk factors. This could imply that primary healthcare is focusing screening on risk groups. However, over 95% of those undiagnosed still belonged to a T2D risk group, defined as having obesity, hypertension, prediabetes, family history of diabetes or being a daily smoker, at the start of the period where they developed T2D. Our study therefore indicates that paying attention to blood glucose levels in risk groups within primary healthcare would lead to the detection of almost all individuals with T2D. However, these risk factors are common in the population, and >76% of those who did not develop T2D also belong to a risk group. We instead suggest defining risk groups as having at least two of the abovementioned factors, which shows a sensitivity and specificity of at least 79% and 59%, respectively. Continuity between the general practitioner and patients is likely to raise awareness of the patients’ risk factors. We encourage further research into the most appropriate definition of risk groups for T2D where screening should be performed. Nevertheless, alternative strategies might be needed to detect all individuals with newly developed T2D.

In agreement with a previous study [16], we found that younger age is associated with not being diagnosed among individuals with T2D. A Norwegian study found that being undiagnosed was associated with having an adverse cardiometabolic profile, including high BMI and hypertension [17] at the time of detection. An Irish study found an association between being undiagnosed and not having a family history of diabetes, having high BMI, and low physical activity at the time of detection [15]. Our analysis examines exposures *prior* to T2D onset and implies the opposite, that having higher BMI and hypertension prior to T2D development increases the probability of being diagnosed among individuals with T2D, and we found no association with physical activity or having a family history of diabetes. This variation between studies may be due to differences in design and risk factor development among individuals or that factors associated with obtaining a diagnosis are healthcare-organization specific.

Previous studies have reported that not regularly visiting a doctor is associated with undiagnosed T2D [16, 18]. We were unable to study healthcare utilization within primary care in our study. However, we found that self-estimated poor general health was associated with the probability of being diagnosed. It seems plausible that individuals who estimate their health as poor might contact healthcare more frequently than those who evaluate their health as good.

In accordance with other European studies [15, 16, 18], we did not find an association between socioeconomic status and being diagnosed by healthcare among individuals with T2D. This could suggest that the current screening does not disadvantage socioeconomic groups in the Swedish population, is due to low power, or reflects participation bias and raised health awareness within the study. Additional studies on the screening practice within healthcare are needed to accurately assess whether screening is equally performed. Previous studies have indicated that socioeconomic status is associated with undiagnosed T2D when compared with individuals with normoglycemia [16, 38, 39], which indicates the known association between T2D susceptibility and lower socioeconomic status [40]. Jointly, these results imply that T2D, independent of whether undiagnosed or diagnosed, is more prevalent among individuals with lower socioeconomic status in Sweden.

### Strengths and limitations

Our cohort provides extensive clinical, biological, and self-reported data from three repeated clinical examinations over 20 years among individuals in Stockholm County. Thanks to register linkage using individual personal identity numbers, we have detailed sociodemographic and health-related information from national and regional registers for the entire cohort, including individuals who dropped out from the clinical examinations.

This study has selection bias towards healthy participants since we only included individuals who were free of T2D at the start of each period. A previous study on SDPP generalizability showed that the baseline study sample has a high generalizability regarding T2D risk [41]. The SDPP clinical cohort only includes individuals born in Sweden, and previous studies have shown a higher T2D risk among individuals born outside of Sweden [42–44], suggesting that the proportion of T2D might be higher among the general population than in our study. The lack of individuals born outside of Sweden also means that we could not investigate a potential association between country of birth and likelihood of being diagnosed. Furthermore, the oversampling of individuals with a family history of diabetes might suggest that we overestimate the proportion of T2D, as family history is a known risk factor for T2D. However, we did not find any association with family history of diabetes regarding the chance of being diagnosed, so that should not limit the generalisability of observed associations with likelihood of T2D being diagnosed. Drop-outs during the study period might further contribute to selection bias. In this study, the participants lost to follow-up between the two periods were more often daily smokers at baseline, less physically active, had more metabolic risk factors, long-term illness, worse general health, and lower socioeconomic status (see Additional file 1: Table S5), similar to what we have previously observed [22]. Jointly, this implies that we might underestimate the proportion of individuals with T2D in our study. Furthermore, undiagnosed T2D could only be assessed among the individuals who attended the OGTT at the follow-ups. Given that higher BMI was associated with dropping out as well as diagnosis status in our study, this suggest that the ratio of undiagnosed to diagnosed T2D might be affected by participation bias. In summary, we assess that our results are broadly generalizable to the Swedish healthcare system. However further studies are needed to determine undiagnosed T2D among individuals born outside of Sweden and younger age groups.

Knowledge on which groups in the population require additional attention to detect T2D at an early stage is important. A strength of our study is that we investigated the characteristics of individuals prior to T2D development and not their characteristics once disease was evident. However, the questionnaire data might still be affected by self-report bias, especially physical activity [45, 46].

Data on T2D diagnosis were collected from inpatient and outpatient care, including primary care. With information from these registers together with OGTTs and self-reported T2D within the SDPP, we could ensure that the diabetes diagnosis was not misclassified as type 1 diabetes and restrict any over- or underestimations of the proportion of T2D. One limitation of the study is that register coverage during period 1 was not as complete as in period 2, which might lead to overestimation of the proportion of undiagnosed T2D during the first period. This bias is limited by the usage of self-reported diagnoses. In this study, we used one OGTT to define T2D status, whereas a confirmatory test is used in the clinical setting.

## Conclusions

Nearly half of individuals who developed T2D during ten years in the study cohort were undiagnosed, and almost everyone who developed T2D belonged to a T2D risk group, regardless of being undiagnosed or diagnosed. Our results thereby indicate the importance of increased screening, and we suggest that screening could be based on the presence of two risk factors. Implementing the recommendations on a maximum number of patients cared for by each general practitioner might facilitate T2D screening through informational continuity.

### Supplementary Information

**Additional file 1: Table S1.** Baseline characteristics* of individuals with diagnosed and undiagnosed T2D during the study. **Table S2.** Association between belonging to a two-factor risk group* and having T2D, undiagnosed T2D, or diagnosed T2D, compared with normoglycemia. **Table S3**. The proportion of diagnosed T2D and the proportion of undiagnosed T2D in sensitivity analyses. **Table S4.** Odds ratios for diagnosed T2D compared to undiagnosed T2D using the 789 complete cases for different risk factors at the beginning of the period. **Table S5.** Baseline characteristics of participants that participated in period 1, period 2 and those lost to follow-up after period 1.

## Data Availability

The SDPP data used for this study are available upon reasonable request, appropriate ethical approval, and data protection arrangements. Data from the SDPP are protected by the General Data Protection Regulation of the European Union, which prevents open access to the personal data of the study. To obtain deidentified data for research purposes, interested researchers can contact the Centre for Epidemiology and Community Medicine in Stockholm, Sweden (ces.slso@regionstockholm.se).

## Abbreviations

ATC: The Anatomical Therapeutic Chemical System
BMI: Body mass index
CI: Confidence interval
ICD-10: The International Classification of Diseases 10^th^ revision
LISA: The Longitudinal Integration Database for Health Insurance and Labour Market Studies
OGTT: Oral glucose tolerance test
OR: Odds ratio
Sd: Standard deviation
SDPP: Stockholm Diabetes Prevention Programme
T2D: Type 2 diabetes
VAL: The Stockholm Regional Healthcare Data Warehouse
