# Supplementary material for: Undiagnosed type 2 diabetes is common – intensified screening of established risk groups is imperative in Sweden: the SDPP cohort
Source: BMC Med. 2024 Apr 19;22:168. doi: 10.1186/s12916-024-03393-0 (PMC11027361; doi:10.1186/s12916-024-03393-0)
Supplement: Supplementary file 1 — Additional file 1: Table S1. Baseline characteristics* of individuals with diagnosed and undiagnosed T2D during the study. Table S2. Association between belonging to a two-factor risk group* and having T2D, undiagnosed T2D, or diagnosed T2D, compared with normoglycemia. Table S3. The proportion of diagnosed T2D and the proportion of undiagnosed T2D in sensitivity analyses. Table S4. Odds ratios for diagnosed T2D compared to undiagnosed T2D using the 789 complete cases for different risk factors at the beginning of the period. Table S5. Baseline characteristics of participants that participated in period 1, period 2 and those lost to follow-up after period 1. [file 12916_2024_3393_MOESM1_ESM.docx]

**Undiagnosed type 2 diabetes is common – intensified screening of established risk groups is imperative in Sweden: The SDPP cohort.**

Hrafnhildur Gudjonsdottir^1,2*^, Per Tynelius^1,2^, Nouha Saleh Stattin^3,4^, Diego Yacamán Méndez^1,2^, Anton Lager^1,2^, Boel Brynedal^1,2^

^1^Centre for Epidemiology and Community Medicine, Stockholm, Sweden

^2^Department of Global Public Health, Karolinska Institutet, Stockholm, Sweden

^3^Academic Primary Healthcare Centre, Stockholm, Sweden

^4^Division of Family Medicine and Primary Care, Department of Neurobiology, Care Sciences and Society, Karolinska Institutet, Huddinge, Sweden

* Corresponding author. Email: hrafnhildur.gudjonsdottir@ki.se

**Table S1.** Baseline characteristics* of individuals with diagnosed and undiagnosed T2D during the study.

|  | **Diagnosed T2D**  **n=515** | **Undiagnosed T2D**  **n=313** |
| --- | --- | --- |
| Sex |  |  |
| Women, % | 46.8 | 44.7 |
| Age (years), mean (Sd) | 54.0(5.8) | 52.5(6.4) |
| BMI (kg/m^2^), mean (Sd) | 29.9(4.6) | 28.4(4.5) |
| Family history of type 2 diabetes, % | 78.5 | 77.6 |
| Highest attained education |  |  |
| University (>12y), % | 25.6 | 23.3 |
| Upper secondary school (12y), % | 46.0 | 52.7 |
| Primary school (9y), % | 28.4 | 24.0 |
| High-and intermediate nonmanual employees, % | 39.5 | 38.1 |
| Fasting plasma glucose (mmol/L), mean (Sd) | 5.6 (0.65) | 5.3 (0.6) |
| Systolic blood pressure, mmHg, mean (Sd) | 138.6 (17.1) | 134.9 (16.3) |
| Daily smoking, % | 26.5 | 28.4 |
| Daily snuff, % | 10.7 | 12.5 |
| Alcohol, g/week, median (25%-75%) | 60.8 (22.7–134.3) | 77.4 (31.4–154.4) |
| Hazardous alcohol consumption, % | 21.6 | 27.2 |
| Physical activity |  |  |
| Sedentary, % | 15.7 | 16.6 |
| Moderate, % | 55.2 | 60.4 |
| Regular, % | 21.8 | 17.6 |
| Active, % | 7.4 | 5.4 |
| Living alone, % | 17.3 | 16.0 |
| Poor general health, % | 5.4 | 2.6 |
| Long term illness, % | 42.2 | 36.7 |

BMI: Body mass index; T2D: Type 2 diabetes

*Collected at the beginning of period 1 or period 2, depending on which period they developed T2D.

**Table S2.** Association between belonging to a two-factor risk group* and having T2D, undiagnosed T2D, or diagnosed T2D, compared with normoglycemia.

|  | Overall incident T2D | Undiagnosed T2D | Diagnosed T2D |
| --- | --- | --- | --- |
|  | OR (95% CI) | OR (95% CI) | OR (95% CI) |
| Period 1 | 7.7 (5.8–10.1) | 5.2 (3.6–7.6) | 10.3 (6.9–15.5) |
| Period 2 | 6.1 (4.8–7.7) | 3.1 (2.2–4.5) | 8.1 (5.9–11.1) |

*Obesity, family history of diabetes, hypertension, daily smoking, or prediabetes

T2D: Type 2 diabetes

Reference: T2D free individuals

**Table S3**. The proportion of diagnosed T2D and the proportion of undiagnosed T2D in sensitivity analyses.

|  | Diagnosed  T2D^*^  % | Diagnosed  T2D^**^  % | Undiagnosed T2D^***^  % | Undiagnosed T2D^**^  % |
| --- | --- | --- | --- | --- |
| Period 1 | 4.3 (336/7820) | 2.3 (180/7820) | 5.3 (313/5867) | 2.7 (157/5867) |
| Period 2 | 11.2 (609/5422) | 6.2 (335/5422) | 12.6 (430/3416) | 4.6 (156/3416) |

* Assuming everyone lost to follow up instead had diagnosed T2D

** Assuming everyone lost to follow up instead had remained and not developed T2D.

*** Assuming everyone lost to follow up instead had undiagnosed T2D

**Table S4.** Odds ratios for diagnosed T2D compared to undiagnosed T2D using the 789 complete cases for different risk factors at the beginning of the period.

|  | **Model A*** | **Model B**** | **Model C***** |
| --- | --- | --- | --- |
|  | OR (95% CI) | OR (95% CI) | OR (95% CI) |
| Sex | 0.93 (0.69-1.24) | - | - |
| Age | 1.04 (1.02-1.07) | - | - |
| Daily smoking | 0.88 (0.64-1.22) | 0.95 (0.69-1.32) | - |
| Daily snuff | 0.79 (0.51-1.24) | 0.83 (0.52-1.33) | - |
| Hazardous alcohol consumption | 0.74 (0.54-1.01) | 0.67 (0.48-0.95) | - |
| Physical activity |  |  | - |
| Sedentary | ref=1 | ref=1 | - |
| Moderate | 0.94 (0.63-1.41) | 0.89 (0.59-1.33) | - |
| Regular | 1.20 (0.74-1.96) | 1.15 (0.70-1.88) | - |
| Active | 1.35 (0.69-2.65) | 1.24 (0.62-2.46) | - |
| Education |  |  |  |
| Primary school (9y) | ref=1 | ref=1 | ref=1 |
| Upper secondary school (12y) | 0.74 (0.52-1.04) | 0.76 (0.54-1.08) | 0.73 (0.51-1.04) |
| University (>12y) | 0.92 (0.61-1.38) | 0.91 (0.60-1.37) | 0.85 (0.55-1.31) |
| High-and intermediate nonmanual employees | 1.05 (0.78-1.40) | 0.97 (0.72-1.31) | 0.93 (0.68-1.26) |
| BMI |  |  |  |
| <25 kg/m^2^ | ref=1 | ref=1 | ref=1 |
| 25-29.99 kg/m^2^ | 1.70 (1.12-2.58) | 1.71 (1.12-2.60) | 1.76 (1.15-2.69) |
| ≥30 kg/m^2^ | 2.57 (1.67-3.97) | 2.63 (1.69-4.07) | 2.70 (1.73-4.23) |
| Family history of type 2 diabetes | 1.07 (0.76-1.52) | 1.11 (0.78-1.59) | 1.13 (0.78-1.62) |
| Hypertension | 1.38 (1.04-1.85) | 1.24 (0.92-1.67) | 1.27 (0.94-1.72) |
| Fasting glucose | 2.24 (1.77-2.83) | 2.15 (1.69-2.73) | 2.21 (1.73-2.82) |
| Living alone | 0.93 (0.63-1.36) | 0.87 (0.59-1.29) | 0.88 (0.60-1.31) |
| Poor general health | 2.11 (0.95-4.71) | 2.26 (1.00-5.10) | 2.35 (1.04-5.31) |
| Long term illness | 1.22 (0.91-1.64) | 1.15 (0.85-1.56) | 1.16 (0.86-1.58) |

*Crude; **Adjusted for sex and age; ***Adjusted for sex, age, daily smoking, daily snuff, alcohol risk consumption and physical activity.

T2D: Type 2 diabetes

Reference: Undiagnosed T2D

**Table S5.** Baseline characteristics of participants that participated in period 1, period 2 and those lost to follow-up after period 1.

|  | The population in period 1 n=7664 | The population  in period 2  n=5148 | Those lost to follow-up n=2516 | P value* |
| --- | --- | --- | --- | --- |
| Sex |  |  |  |  |
| Women, % | 61.1 | 60.0 | 63.4 | 0.004 |
| Age (years), mean (Sd) | 47.0(4.9) | 47.0(4.9) | 47.0(5.1) | 0.84 |
| BMI (kg/m2), mean (Sd) | 25.6(3.9) | 25.3(3.6) | 26.2(4.5) | <0.001 |
| Family history of type 2 diabetes, % | 57.7 | 57.8 | 57.3 | 0.68 |
| Highest attained education |  |  |  |  |
| University (>12y), % | 33.5 | 34.7 | 31.1 | <0.001 |
| Upper secondary school (12y), % | 47.9 | 48.4 | 47.0 |  |
| Primary school (9y), % | 18.6 | 17.0 | 21.9 | <0.001  <0.001  <0.001 |
| High-and intermediate nonmanual employees, % | 43.9 | 46.2 | 39.0 |  |
| Fasting plasma glucose (mmol/L), mean (Sd) | 4.7(0.54) | 4.7(0.50) | 4.8(0.59) |  |
| Systolic blood pressure, mmHg, mean (Sd) | 122.6(15.8) | 121.7(14.9) | 124.3(17.2) | <0.001 |
| Daily smoking, % | 26.0 | 23.0 | 32.1 | <0.001 |
| Daily snuff, % | 7.8 | 7.8 | 7.8 | 0.95 |
| Alcohol, g/week, median (25%-75%) | 46.6 (18.2-91.6) | 47.5(19.1-92.2) | 44.6(16.6-89.7) | 0.01 |
| Physical activity |  |  |  |  |
| Sedentary, % | 10.7 | 9.6 | 12.8 | <0.001 |
| Moderate, % | 54.7 | 53.5 | 57.0 |  |
| Regular, % | 26.9 | 28.8 | 22.8 |  |
| Active, % | 7.8 | 8.0 | 7.4 |  |
| Living alone, % | 16.3 | 14.8 | 19.5 | <0.001 |
| Poor general health, % | 2.0 | 1.7 | 2.7 | 0.001 |
| Long term illness, % | 27.7 | 26.3 | 30.5 | <0.001 |

BMI: Body mass index; T2D: Type 2 diabetes

*Between those followed in period 2 and those lost to follow-up from period 1

P value: Independent t test, Wilcoxon rank-sum test, Chi2 test
